# *In-vivo* Intramuscular Collagen Synthesis, Muscle Fiber Growth and Histomorphology of *Pectoralis major* of a Fast-Growing Broiler Strain *Gallus gallus domesticus*

**DOI:** 10.3389/fvets.2019.00470

**Published:** 2020-01-10

**Authors:** Pramir Maharjan, Casey M. Owens, Craig Coon

**Affiliations:** Department of Poultry Science, Center of Excellence for Poultry Science, University of Arkansas, Fayetteville, AR, United States

**Keywords:** broiler, *Pectoralis major*, collagen, fractional synthesis rate, fractional degradation rate, collagen turnover, fiber growth

## Abstract

Collagen protein has been considered as major culprit to myopathy condition affecting *Pectoralis major*, called woody breast (WB) in *Gallus gallus domesticus* (broiler). The WB myopathy is characterized by macroscopic stiffness of *P. major* and the affected tissue have reduced protein quality. This study measured the *in-vivo* soluble (S-) and insoluble (I-) collagen fractional synthesis and degradation rates (FSR and FDR) in *P. major* over typical grow-out cycle of broiler using stable isotope of 1-^13^C proline as metabolic tracer. Collagen content and muscle fiber histomorphology of *P. major* were also assessed simultaneously. The FSR and FDR for S- and I-collagen decreased over age, however FSR remained higher than FDR suggesting collagen was accreting during the grow-out period. This was reflected by increment in total collagen content in *P. major* in maturing broiler. Histomicrographs showed myodegeneration occurring as early as 21 days followed by greater accumulation of collagenous tissue in perimysial and endomysial connective tissue spaces of muscle fibers as bird aged. The findings suggest that reduced turnover of collagen in *P. major* at the later age of bird could have evolved due to adaptive physiological feedback mechanism against further synthesis and deposition of collagen in the extracellular matrix.

## Introduction

The global poultry industry ventures to produce larger broilers with higher breast yield in a curtailed growth cycle. This is attained through intensive genetic selection and improved nutritional regimes that support the genetics of these high yielding broiler strains. Selection for quantitative traits such as breast yield and growth rate exert physiological pressure leading to ante-mortem histological and biochemical alterations in muscle tissues. The industry has recently witnessed a myopathy condition affecting *Pectoralis major* (breast muscle) of broilers, called woody breast (**WB**), an issue subject to both economic losses and welfare of birds. A recent study reported WB myopathy incidence of ~9% for 10,483 filets evaluated in high breast meat yielding strain from a flock which produced larger broilers (2.72–4.53 kg) (1). The WB myopathy condition is mainly characterized by macroscopic stiffness in breast tissue affecting the appearance and protein quality (2, 3). Histologically, it exhibits moderate to severe polyphasic myodegeneration with variable degrees of interstitial connective tissue accretion or fibrosis (4).

Understanding possible biological mechanisms and pathways associated with the onset and progression of WB myopathy can be crucial to minimizing the incidence of muscle myopathy. The exact etiology for WB myopathy is still unclear to scientific community, even though proteomic changes related to carbohydrate and protein metabolism have been suggested. Of many proteins potentially involved in WB myopathy, collagen is considered the key protein involved in WB myopathy condition in *P. major* (2–5). Collagen is an extracellular connective tissue matrix in muscle fibers that forms collagen fibrils, which then crosslink to give stiffness to muscle tissues (6). The arrangement types and extent of cross-linking between collagen fibrils can vary between broiler lines (7). Since quantitative intramuscular collagen availability, and degree of crosslinking between the collagen fibrils are intrinsic histo-biochemical phenomena that determine WB myopathy condition, the current study attempts to uncover collagen protein turnover (**PT**) occurring during the broiler grow-out cycle. Fractional synthesis rates (**FSR**) and fractional degradation rates (**FDR**) were measured for insoluble (**I-**) and soluble (**S-**) collagen. The stable isotope method was utilized in this study to understand collagen PT changes occurring in *P. major* muscle. The stable isotope, 1-^13^C proline was used as a metabolic tracer and was infused in the birds' circulatory system via brachial vein utilizing flooding technique. The measurement of *in vivo* collagen synthesis using stable isotope methodology is novel to broiler species and elaborated discussions made on the method and result sections in present study can potentially aid in application of this method to other avian species to measure collagen synthesis. Understanding collagen turnover in *P. major* during the broiler grow-out cycle can contribute to the development of nutritional and management strategies to modulate collagen biosynthesis that can subsequently aid in minimizing incidence of WB myopathy. Along with PT changes occurring in a typical grow-out phase of broiler, collagen quantification, and fiber growth in *P. major* were also assessed simultaneously in this study.

## Methods

### Bird Type and Husbandry

The experiment was conducted in a fast-growing commercial meat type broiler, Cobb 700, *Gallus gallus domesticus*, which were fed diets (Table 1) *ad libitum* as per industry guidelines (Broiler Performance and Nutrition Supplement, 2012). Birds (*n* = 900) were placed in 36 pens, 25 birds per pen (pen size of 2.006 m^2^) and were reared from 0 to 57 days.

**Table 1:** Experimental diets^*^.

**Ingredients**	**Starter** ** 1–10 days (%)**	**Grower** ** 11–20 days (%)**	**Finisher I** ** 21–42 days (%)**	**Finisher II** ** 43–57 days (%)**
Yellow corn 7.4%	45.46	52.68	60.30	62.29
Soybean meal, 44%	42.52	34.62	27.04	25.43
Proplus	4.73	6.13	6.04	5.86
Poultry fat	4.00	4.00	4.00	4.00
Limestone	0.31	0.04		
Dicalcium phosphate	1.11	0.79	0.71	0.60
Salt	0.54	0.46	0.46	0.44
Methionine 98.5%	0.43	0.40	0.46	0.38
Lysine	0.23	0.24	0.40	0.33
Choline chloride-60	0.15	0.15	0.15	0.15
Vitamin premix^2^	0.10	0.10	0.10	0.10
Mineral premix^2^	0.10	0.10	0.10	0.10
Threonine 98%	0.23	0.20	0.26	0.23
Selenium premix 0.06%	0.02	0.02		
Monsanto Santoquin ethoxyquin	0.02	0.02	0.02	0.02
Mold curb	0.05	0.05	0.05	0.05
**Calculated composition**				
True Metabolizable energy (Kcal/kg)	3,000	3,100	3,191	3,215
Crude protein	26.4	24	21.12	20.38
Crude fat	6.88	7.61	7.31	7.33
Calcium	1	0.90	0.9	0.85
Phosphorous, non-phytate	0.5	0.48	0.45	0.45
Digestible arginine	1.65	1.46	1.24	1.18
Digestible lysine	1.46	1.29	1.18	1.14
Digestible methionine	0.755	0.69	0.66	0.63
Digestible methionine and cysteine	1.08	0.98	0.92	0.88
Digestible leucine	1.88	1.71	1.52	1.48
Digestible isoleucine	0.99	0.87	0.73	0.70
Digestible threonine	1.08	0.96	0.91	0.86
Digestible valine	1.07	0.96	0.83	0.80
**Analyzed composition**				
Crude protein, %	25.3	23.2	20.4	19.9
Calcium, %	1.09	1.05	0.94	0.83
Total phosphorus, %	0.72	0.71	0.69	0.64

* *Supplies per kilogram of diet: antioxidant, 200 mg; retinyl acetate, 21 mg; cholecalciferol, 110 μg; D-α-tocopherol acetate, 132 mg; menadione, 6 mg; riboflavin, 15.6 mg; D-calcium pantothenate, 23.8 mg; niacin, 92.6 mg; folic acid, 7.1 mg; cyanocobalamin, 0.032 mg; pyridoxine, 22 mg; biotin, 0.66 mg; thiamine, 3.7 mg; choline chlorine, 1,200 mg; Mn, 100 mg; Mg, 27 mg; Zn, 100 mg; Fe, 50 mg; Cu, 10 mg; I, 1 mg; Se, 200 μg*.

### Sampling for Collagen Protein Turnover Study, Histo-Morphology, Collagen Quantitation, and Shear Force Measurement

Thirty-five broilers were selected at each sampling age within the weight range of mean pen weight (±1 SD). Broilers were selected from seven pens for each sampling occasions at d 21, 28, 35, and 42, and from eight pens on d 57 age of bird. The broiler pens utilized for selection at each sampling age were then excluded from further selections during the study. Twenty broilers (*n* = 10 infused at each sampling age) were utilized for collagen PT study (methods discussed below in details) in *P. major* muscle. Infused broilers at d 35, 42 and 57 were so selected and grouped as myopathy [*n* = 5 toward myopathy (WB score ≥ 1)] and non-myopathy [*n* = 5 toward non-myopathy (WB score ≤0.5)]. Breast muscle tissue samples were initially collected (right-half of filet), and left half of the filet from each sampled broiler was then subjectively scored (d 35, 42, and 57) for WB myopathy condition (8). Tissue samples from broilers utilized for PT study were also used for *P. major* fiber growth study, collagen quantitation, and SF measurement. Shear force was measured in all 35 birds selected utilizing one-half (left) of breast filet (hot deboned) (8). The SF values obtained were correlated with weight and height of filet.

Fiber growth of *P. major* was determined at six different ages (d 21, 28, 35, 42, 49, and 57) during the broiler grow-out cycle, whereas five different ages—d 21, 28, 35, 42, and 57 were utilized for SF measurements, collagen quantitation, histomorphology, and *in-vivo* collagen synthesis.

### Measurement of *in-vivo* Collagen Turnover Using Stable Isotope Tracer Technique

#### Isotope Preparation and Infusion

L-proline-1-^13^C (Sigma Aldrich, 81202-06-4) isotopic solution at 20 atomic percent excess (APE) was prepared fresh (1 h before the infusion) during each sampling age and each broiler sampled was infused with the solution through brachial vein at the rate of 10 ml/kg BW with 1 min interval (flooding dose). Non-infused birds (*n* = 10) were treated as control. Control birds were scanned for body composition using dual energy x-ray absorptiometry (DEXA) equipped with Lunar Prodigy small animal software to determine body composition of sampled birds used in PT study (Table 2).

**Table 2:** Representative body composition of birds used for collagen protein turnover at each sampling age^*^.

	**Live BW**	**Lean mass (g)**	**Fat mass (g)**	**Energy (kcal)**	**Mineral (g)**	**Ca (g)**	**P (g)**
d 21	894 ± 26.59	793.42 ± 23.51	49.84 ± 4.71	1, 359.74 ± 49.17	18.69 ± 0.52	3.96 ± 0.11	3.28 ± 0.09
d 28	1, 463.33 ± 37.49	1, 288.39 ± 25.34	120.07 ± 5.26	2, 504.08 ± 80.30	33.6 ± 1.08	7.38 ± 0.25	6.01 ± 0.19
d 35	2, 133 ± 39.47	1, 676.49 ± 27.09	322.36 ± 5.73	4, 463.12 ± 120.06	51.83 ± 1.41	11.68 ± 0.33	9.39 ± 0.26
d 42	2, 953.5 ± 58.72	2, 569.21 ± 54.88	282.62 ± 11.99	5, 448.14 ± 126.38	63.69 ± 1.52	14.53 ± 0.36	11.61 ± 0.28
d 49	3, 471 ± 23	3, 029.71 ± 12.03	326.1 ± 2.66	6, 414.61 ± 122.05	69.17 ± 8.01	15.86 ± 0.87	12.64 ± 0.67
d 57	4, 087.4 ± 103.94	3, 568.69 ± 106.21	380.97 ± 23.71	7, 585.41 ± 202.11	80.53 ± 3.15	18.64 ± 0.76	14.79 ± 0.59

* *Body composition was studied using dual energy x-ray absorptiometry (DEXA); Ca, Calcium; P, Phosphorus; n = 10*.

#### Blood and Tissue Sample Collection

**Figure 1:**
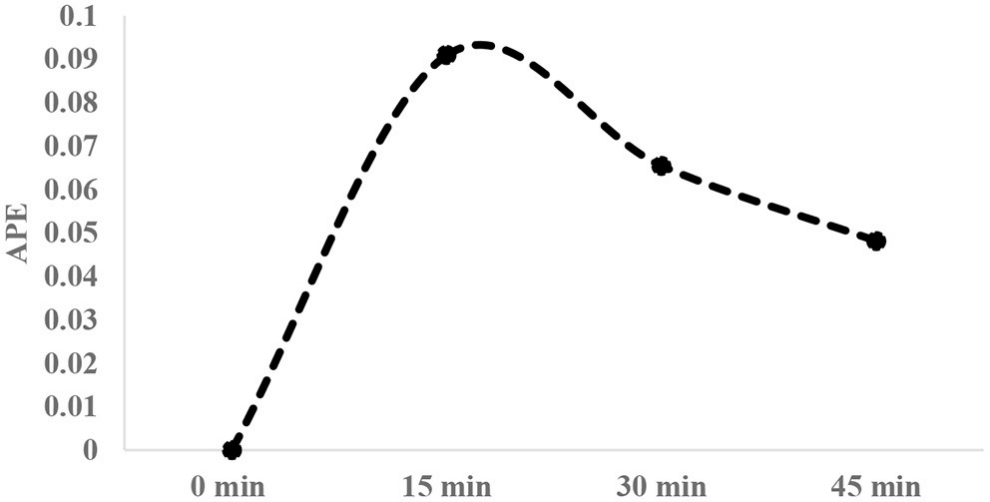
Typical enrichment decay of 1-^13^C proline in plasma over 45-min interval post infusion of tracer [20 atom percent excess (APE)].

After infusion of isotopic solution, arterialized venous blood was collected (1–2 ml) at three different occasions (15 min interval), using brachial vein and broilers were then sacrificed to collect breast tissue samples. Average enrichment decay of free 1-^13^C proline in plasma over 45 min time period post isotopic infusion was measured (Figure 1). Breast tissue (~150 g wet tissue) (cranio-ventral portion) samples were collected and immediately frozen in liquid nitrogen and stored at −80°C until analysis. Plasma was separated from the blood and stored similarly at −80°C until analysis.

Blood and breast tissue samples from control birds (*n* = 10) were similarly collected for all sampling ages to understand the baseline enrichment of 1-^13^C proline.

#### Determination of Tissue and Plasma Enrichment in Infused Birds

Collagen protein fractions S-collagen and I-collagen were separated as described elsewhere (9, 10). Briefly, tissue samples were lyophilized, ground to powder and added to homogenization buffer with protease and phosphatase inhibitor (0.15 M NaCl, 0.1% Triton X-100, 0.02 M Tris-HCl buffer, 5 mM EDTA, pH = 7.4). The tissue solution was incubated for 3 h. The solution was then sequentially centrifuged (1600× g, 20 min, and 4°C) to separate out collagen fraction. The collagen protein fraction was subjected to overnight incubation with 0.1% pepsin and 0.5 M acetic acid and centrifuged to separate the precipitated pellet (I-collagen fraction). The S-collagen was precipitated from the supernatant fraction following centrifugation using salt (0.9 M NaCl). The protein fractions (I-collagen and S-collagen) were acid hydrolyzed (6 M HCl) for 24 h at 110°C to release protein-bound amino acids and then purified using Dowex 50W-X8 H^+^ ion-exchange resin. Purified amino acids (including proline) were derivatized with N-methyl-N-tert-butyldimethylsilyltrifluoroacetamide (Sigma, CAS Number 77377-52-7) to tert-butyldimethylsilyl (t-BDMS) compounds to get tBDMS-proline. Derivatized samples were subjected to gas chromatography-mass spectrometer (GC MS) (Agilent Technologies, Santa Clara, CA) analysis and the mass spectrum (ion fragmentation pattern) of tBDMS-proline was obtained.

**Figure 2:**
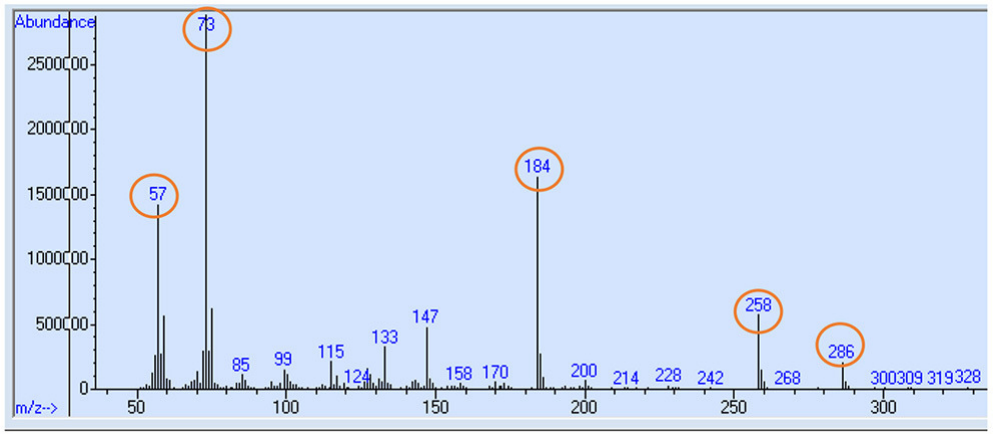
Typical mass chromatogram ion fragmentation pattern of tBDMS-proline produced from plasma or tissue samples in GC MS. Selected ion monitoring for mass to charge ratio (m/z) ratio of 73, 147, 184, 258, and 286 (highlighed in circle) were performed, and ratio of 287–286 was taken to measure the ^13^C-proline enrichment.

Free amino acids including proline from plasma samples were isolated and proline was derivatized to tBDMS-proline before the mass spectra from GC MS were generated. Selected ion monitoring was performed for mass to charge ratio (m/z) ratio of 73, 147, 184, 258, and 286 (11). Typical mass spectrum ion fragmentation pattern of tBDMS-proline produced is given in Figure 2. Enrichment (E), which is the isotopic ^13^C-to-^12^C ratio, was measured using the ratio of fragments 287–286. Enrichment was then adjusted to APE values.

#### Determination of Fractional Synthesis Rate (FSR) for Collagen

FSR was determined using traditional precursor–product principal (12), which is given by:

$$\begin{array}{l} \text{FSR}\left(\text{\%}{\text{h}}^{-1}\right)=\Delta \text{Ep}/\text{A}\times 1/\text{t}\times \;100 \end{array}$$

Where “ΔEp” is the difference in enrichment of 1-^13^C proline measured in terms of APE between infused and control (basal) tissue samples. Therefore,

$$\begin{array}{l} \Delta \text{Ep}=\text{Ei}-\text{Ec} \end{array}$$

Where Ei = enrichment of tissue or plasma sample in infused birds.

Ec = enrichment (baseline) of tissue or plasma in control birds.

A is the average enrichment of tracer (1-^13^C proline) determined as the area under the time-plasma enrichment curve, and t is the time (in hours) of tracer (1-^13^C proline) incorporation in product.

#### Determination of Fractional Degradation Rate (FDR) for Collagen

Growth rate on per day basis for collagen tissue, for S-collagen and I-collagen, was measured by quantifying collagen from collected breast tissues samples at each sampling occasion. The degradation rate (determined on a per day basis) was measured and converted to per hour basis. The fractional growth rate (FGR) and fractional degradation rates (FDR) were expressed as follows: FGR = {[Weight of protein fraction (W2) at time t1—weight of protein fraction (W1) at time t2]/[Weight of protein fraction (W2) at time t1]} × 100.

Weight of collagen protein fractions were measured using hydroxyproline (HP) assay (methods explained below). The FDR was measured using the following equation: FDR (% h^−1^) = FSR–FGR.

FSR and FDR values were obtained for individual broiler at each sampling occasion, and average values were determined. The percent accretion or turnover (% d ^−1^) of S- and I-collagen were then measured by the difference of average values of FSR and FDR for each sampling age. Also, average FSR values were separately determined for d 35, 42, and 57 as myopathy (WB score ≥1) and non-myopathy (WB score ≤ 0.5).

### Tissue Collagen Quantitation

For the collected breast tissue samples (mid-ventral region, *n* = 10 birds), collagen was quantified using HP assay (13). Briefly, separated S-collagen and I-collagen fractions were lyophilized, acid hydrolyzed for 24 h using 6 M HCL (5 mg/ml) at 115°C, and then neutralized with base. The sample solution was mixed with buffered chloramine T reagent (5 ml of choramine T solution, then diluted with 7.5 ml of n-propanol and 12.5 ml of acetate citrate buffer). Ehrich's reagent [2 g of 4-(dimethylamino) benzaldehyde (Sigma Aldrich product #156477) in 3 ml of 60% (v/v) perchloric acid mixed with 13 ml of n-propanol] was added to the solution mixture, and the solution was incubated at 60°C for 20 min. Following incubation, absorbance of the solution was measured at 550 nm to determine the HP concentration. Standard curves of trans-4-hydroxy-L proline (Sigma Aldrich Product # H54409) were produced to find the unknown concentration of HP in sample solutions. Average collagen content for all sampling ages were determined, and collagen content for non-myopathy and toward myopathy broilers were separated for ages d 35, 42, and 57.

### Histomorphology

Breast muscle tissue from mid-ventral region (*n* = 10 per age group of PT sampling occasions) from 21 to 57 days were evaluated for histo-morphology. Collected tissue samples were fixed in 10% formalin solution. Formalin fixed tissue was subjected to histological slide preparations for Masson Trichome (MT) staining. Images were captured using light microscopy and visualized on a color monitor hooked to Image Proplus software. For improving contrast in MT stained slides (d 42 myopathy), confocal microscopy (Leica, CTR 6500) was also utilized. Further, the fibrous nature of collagen in affected muscle tissue were confirmed by ultra-images taken by scanning electron micrograph (ESEM, XL 30). Micrographs captured were studied for the muscle fiber characteristics and relative myo-degeneration. Average fiber diameter and fiber number per unit area were determined for approximately 100 fibers taking four random fields for each histological slide. Non-fiber space was estimated by subtracting total muscle fiber area from mean cross-sectional area (within muscle fascicle) used for assessing the muscle fiber number and diameter.

### Data Analysis

The data obtained was analyzed by one-way ANOVA using JMPro 14 (SAS Institute, Inc., Cary, NC). Mean values were obtained for variables measured (FSR, FDR, % accretion, collagen content, and fiber growth characteristics) for different sampling ages. Significant means for the measured variables were separated using student's *t*-test or HSD test where appropriate. Means were considered significant for *P* ≤ 0.05. The multivariate correlation values were calculated for filet height, SF and body weight of sampled broilers.

## Results

### Collagen Protein Turnover

**Figure 3:**
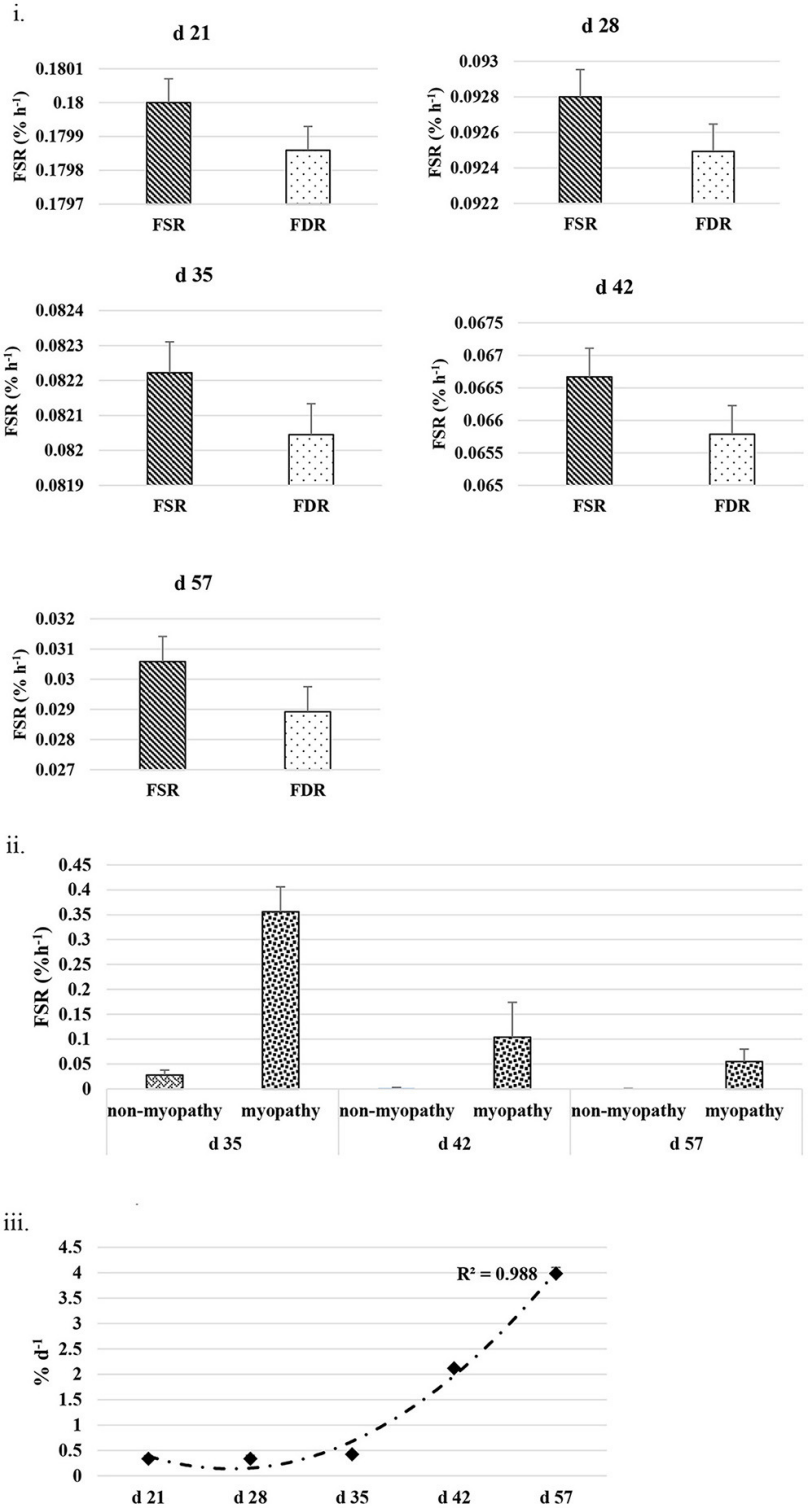
**(i)** Average fractional synthesis rates (FSR) and fractional degradation rates (FDR) for insoluble collagen in *Pectoralis major* at five different ages (*P* < 0.05 between ages) of bird grow-out cycle. The FSR values for d 35, d 42, and d 57 in <30% (*n* ≤ 3 birds) of sampled birds (*n* = 10) showed greater individual variation. Therefore, those values were excluded while constructing graphs, however those individual variation in FSR values were discussed in the result section. **(ii)** Fractional synthesis rates for myopathy (WB score ≥1) and non-myopathy birds (WB score ≤0.5). **(iii)** Percent accretion (%d^−1^) for insoluble collagen in *P. major* at five different ages of bird grow-out cycle.

**Figure 4:**
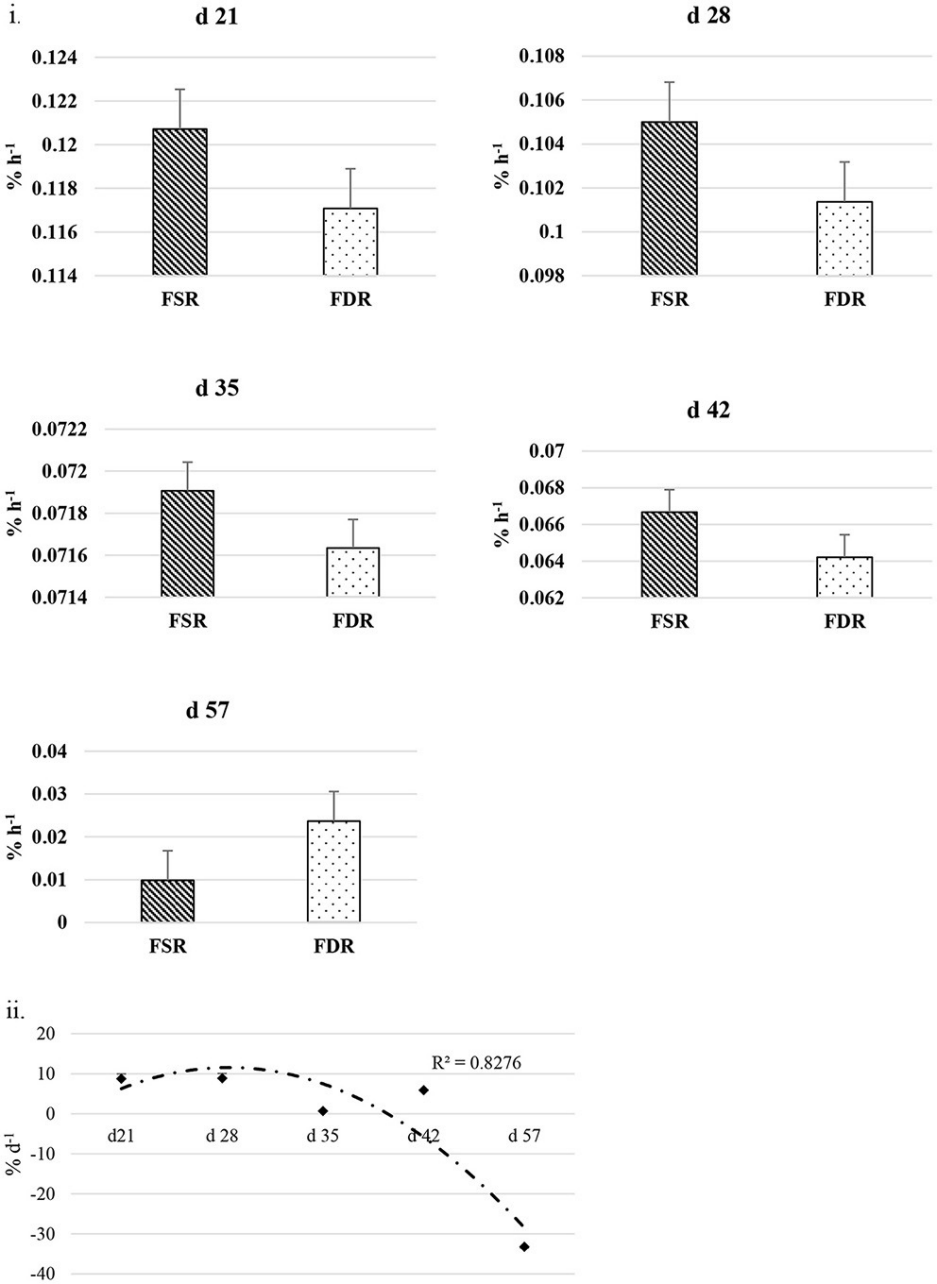
**(i)** Fractional synthesis rates (FSR) and fractional degradation rates (FDR) for soluble collagen in *Pectoralis major* at five different ages (*P* < 0.05 between ages) of bird grow-out cycle. The FSR values for d 35, d 42, and d 57 in <30% (*n* ≤ 3 birds) of sampled birds (*n* = 10) showed greater individual variation. Therefore, those values were excluded while constructing graphs, however those individual variation in FSR values were discussed in the result section. **(ii)** Percent accretion (% d^−1^) for soluble collagen in *Pectoralis major* at five different ages of bird grow-out cycle.

The FSR and FDR for I-collagen and S-collagen at various ages are given in Figures 3i, 4i, respectively. The average FSR and FDR values for I-collagen at d 21 were 0.180 ± (0.033) % h^−1^ and 0.179 ± (0.03) % h^−1^, respectively. The FSR and FDR for I-collagen decreased from 21 to 57 days of age. The FSR and FDR for I-collagen in *P. major* for d 57 broilers were 0.03 (± 0.009) % h^−1^ and 0.0289 (±0.009) % h^−1^, respectively. For S-collagen at d 21, the FSR was 0.120 (± 0.017) % h^−1^ and FDR was 0.117 (± 0.016) % h^−1^. The FSR and FDR of S-collagen tended to decrease with increasing age of the broiler. The FSR was higher than the FDR until d 42 for S-collagen. At d 57, the S-collagen exhibited higher FDR value than FSR (0.023 vs. 0.009% h^−1^).

The FSR values for S-collagen or I-collagen at d 35 or higher sampling ages had greater individual variation for some replicate samples (≤30% of sample size). Samples that measured higher values of FSR at d 35 were 0.17, 0.20, and 0.46% h^−1^ for I-collagen, and 0.10 and 0.29% h^−1^ for S-collagen. At d 42, two of the replicates (of *n* = 10) had FSR of 0.13 and 0.39% h^−1^ for I-collagen and one bird (of *n* = 10) had FSR for S-collagen of 0.46% h^−1^. One broiler (of *n* = 10) had FSR value 0.21% h^−1^ for I-collagen. When FSR values for non-myopathy and myopathy broilers were considered separately for each age, higher FSR for I-collagen was occurring for myopathy broilers (Figure 3ii).

The FSR values were higher than FDR values for I-collagen and this difference between FSR and FDR values became greater at succeeding sampling ages. The percent accretion (% d^−1^) of I-collagen was ~0.5% d^−1^ at d 21, which then increased to almost 4 % d^−1^ at d 57 (Figure 3iii) as explained by polynomial regression curve (*R*^2^ = 0.98). The data for S-collagen indicated that accretion rate was positive from 21 to 42 days, followed by negative accretion at d 57 (*R*^2^ = 0.8276) (Figure 4ii).

### Collagen Quantitation

**Figure 5:**
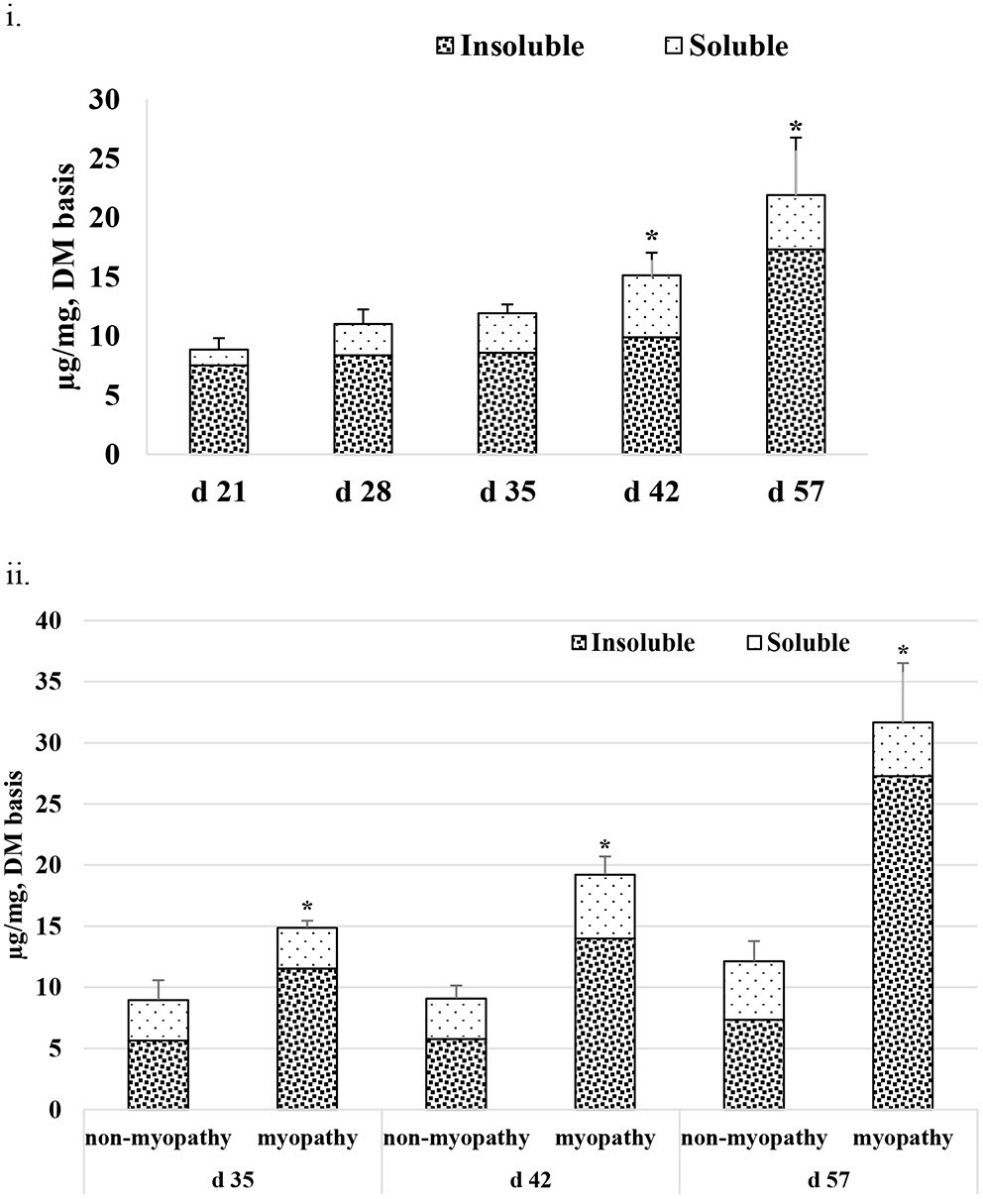
Average total collagen as shown by soluble and insoluble collagen fractions (DM basis) at various ages (21–57 days) of bird grow-out cycle. **(ii)** Collagen content for myopathy (WB score ≥1) and non-myopathy birds (WB score ≤0.5) for d 35–57. Asterisk (*) on top of bar represents the significantly different (*P* < 0.05) amount of insoluble collagen between **(i)** and within ages **(ii)**.

Figure 5i gives the average total collagen content detected *in P. major* at different ages of the sampled broiler during the grow-out cycle and is presented in the form of I-collagen and S- collagen fractions on a dry matter basis. Total collagen increased linearly (*R*^2^ = 0.84) from 8.85 (±0.96) μg/mg to 21.89 (±4.86) μg/mg. The amount of I-collagen at d 21 was 7.52 μg/mg of total collagen. As bird aged, the amount of I-collagen in the muscle increased (*P* < 0.05) to 17.31 μg/mg of the total collagen. S-collagen at d 21 was 1.33 μg/mg and increased to 5.22 μg/mg at d 42, and then decreased to 4.38 μg/mg at d 57. When collagen content was compared between non-myopathy and myopathy groups in *P. major*, it was higher (*P* < 0.05) for myopathy affected broiler for d 35, 42, and 57 (Figure 5ii).

### Fiber Growth

**Figure 6:**
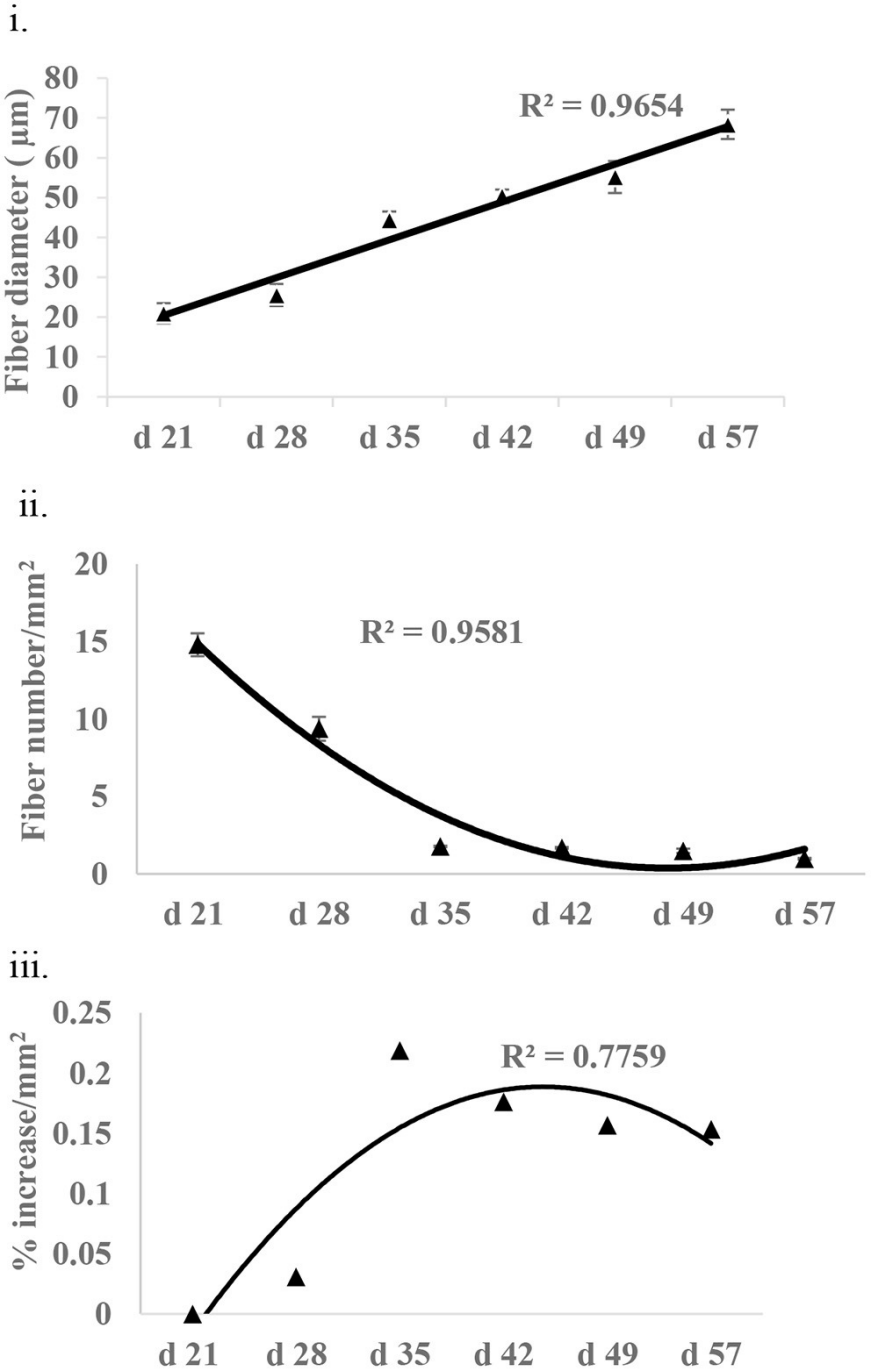
Fiber growth at various ages (21–57 days) of bird as assessed by **(i)** fiber diameter **(ii)** fiber number, and **(iii)** non-fiber space (% increase in non-fiber space per mm^2^ of tissue as compared to non-fiber space available at d 21).

Fiber growth was assessed in the form of fiber diameter, fiber number, and non-fiber space in *P. major* at various ages of bird grow-out cycle (Figures 6i–iii). Fiber diameter at d 21 was 20.97 (±2.52) μm and then increased linearly (*R*^2^ = 0.96) to 68.39 (±6.68) μm by d 57. Similarly, fiber number at d 21 was 1,479.05 (±73.83) per mm^2^ and decreased quadratically (*R*^2^ = 0.95) to 97.88 (±5.13) per mm^2^ of muscle tissue at d 57. Non-fiber space at d 28 as compared to d 21 was 3% higher per mm^2^ of muscle tissue and tended to increase quadratically to 15% or more per mm^2^ from d 35 to 57.

### Histomorphology

**Figure 7:**
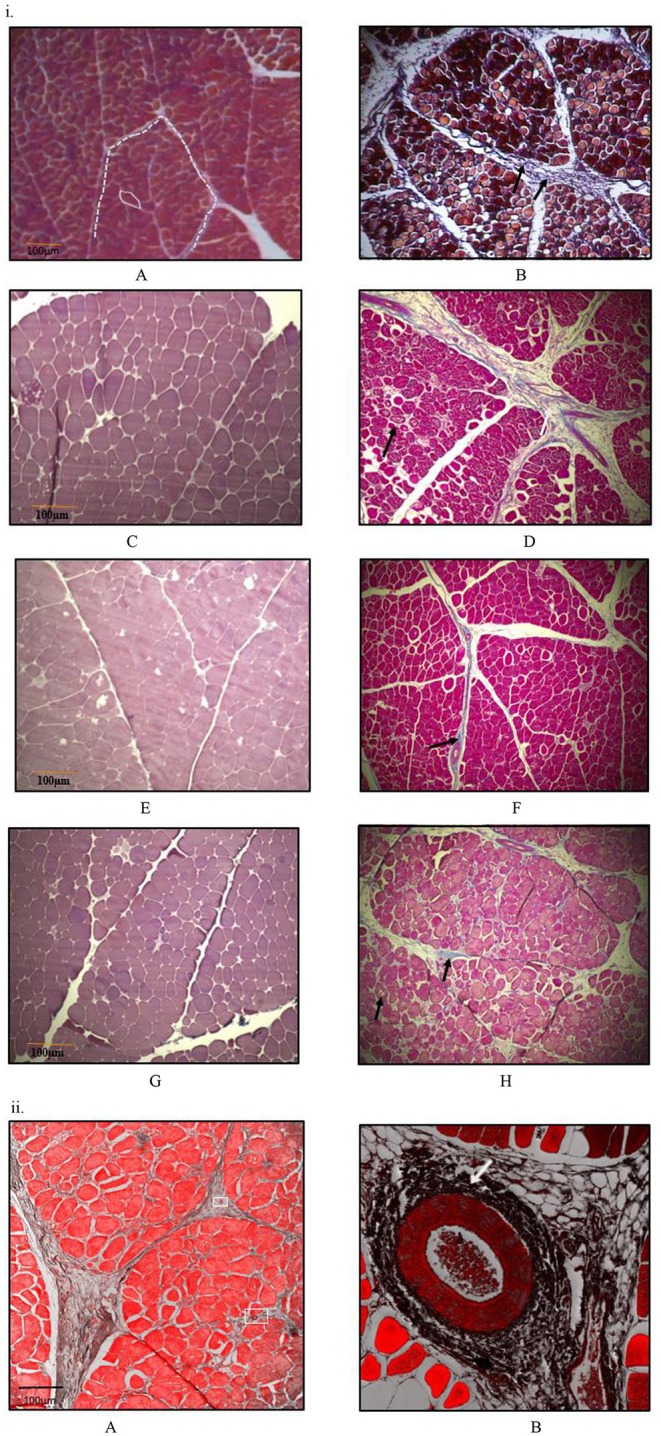
Histomicrographs (Masson-Trichome staining) of cross-section of *Pectoralis major*. **(i)** Micrographs presented on left side (A,C,E,G) have less myodegeneration (non-myopathy birds) occurring in muscle fibers than micrographs (myopathy birds) presented on right side (B,D,F,H) for bird age of days- 21, 35, 42, and 57. Micrograph A: day 21 micrograph demonstrating tightly packed polygonal fibers with a minimal extracellular connective tissue space. White solid line outline represents the endomysial connective tissue space, and dashed line represents perimysial tissue space. Micrograph B: day 21 micrograph with wider perimysial region, and collagen deposition in perimysial region (indicated by arrow). (C,D) Day 35 micrographs; degenerating muscle fibers are elsewhere in micrograph D as indicated by black arrow and wider perimysial space filled with collagenous tissue (indicated by white arrow). (E,F) Day 42 micrographs; Perimysial and endomysial connective tissue spaces are wider in (F) as compared to (E) and the spaces are filled with collagenous tissue. Perivascular infiltration of immune cells was present around the blood vessels in (F) [magnified pic: **(ii)**]. (G,H) day 57 micrographs; Perimysial and endomysial connective tissue spaces filled with collagenous tissue [bluish coloration in (H)]. Greater variation in shape and size of fibers were observed in (H). Significant necrotic patches (white areas indicated by arrow) were also observed in degenerating fibers in (H). A tight parallel organization of perimysial collagen fibrils (indicated by arrow) are occurring as seen in (H) suggesting higher degree of collagen cross-linking. **(ii)** Histomicrographs of cross-section of *P. major* muscle at d 42 from a myopathy bird. Confocal images were obtained to improve contrast between vein, infiltrating cells and myofibers. (A) Thick perimysial collagenous tissue and fibrosed veins (indicated within white boxes) seen in connective tissue spaces leading to atrophy of peripheral fibers. (B) Magnified image of a fibrosed vein with perivascular infiltration of densely packed immune cells (indicated by white arrow).

**Figure 8:**
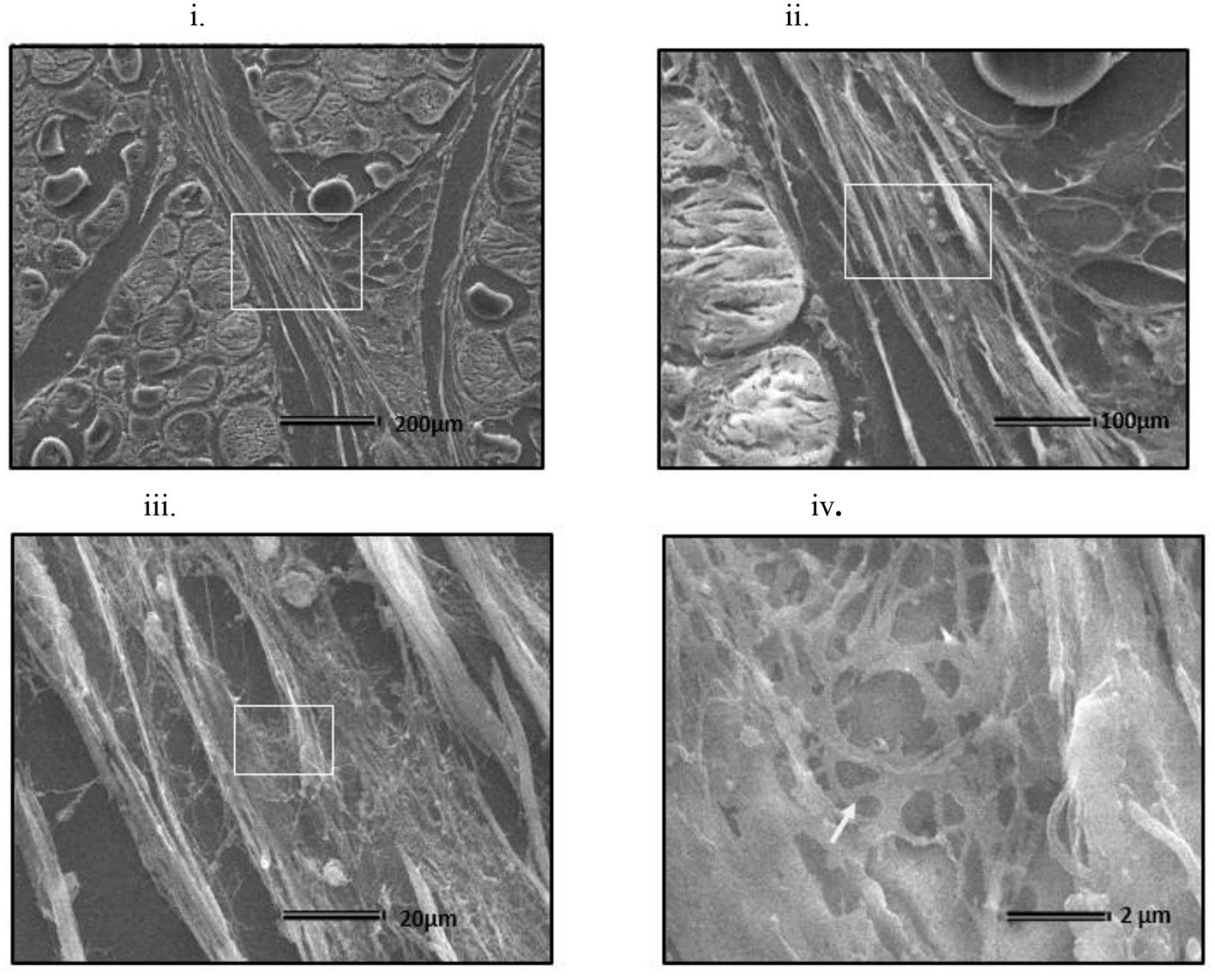
Scanning electron micrograph of cross section of myopathy affected *Pectoralis major* muscle **(i)** Collagen fibrils making collagen fiber bundles (bundle width measured up to several μm in diameter) were evident and were tightly arranged across longitudinally in perimysial spaces. White arrows on image **(i)** indicates degenerating muscle fiber. White rectangular area on image **(i)** were sequentially magnified to get images **(ii–iv)** to obtain ultrastructure entangled collagen fibrils in image **(iv)** (indicated by white arrow).

At all sampling ages, varied degrees of myodegeneration in *P. major* muscle was observed. Myodegeneration of muscle fibers were evident starting at as early as d 21 (Figure 7i). Figure 7 depicts histomicrographs for normal vs. myopathy-affected broilers at d 21, 35, 42, and 57. As myodegeneration increased, which was evident as the broiler aged, there was relatively more accumulation of collagenous tissue (blue coloration upon MT staining) in perimysial and endomysial connective tissue space. Perivascular infiltration of immune cells in perimysial and endomysial spaces were also evident in myopathy-affected broiler (Figure 7ii). Further observation utilizing scanning electron (SE) images in myopathy-affected tissue showed collagen fiber bundles each of several μm in diameter arranged tightly in perimysial connective space (Figure 8).

### Shear Force

**Figure 9:**
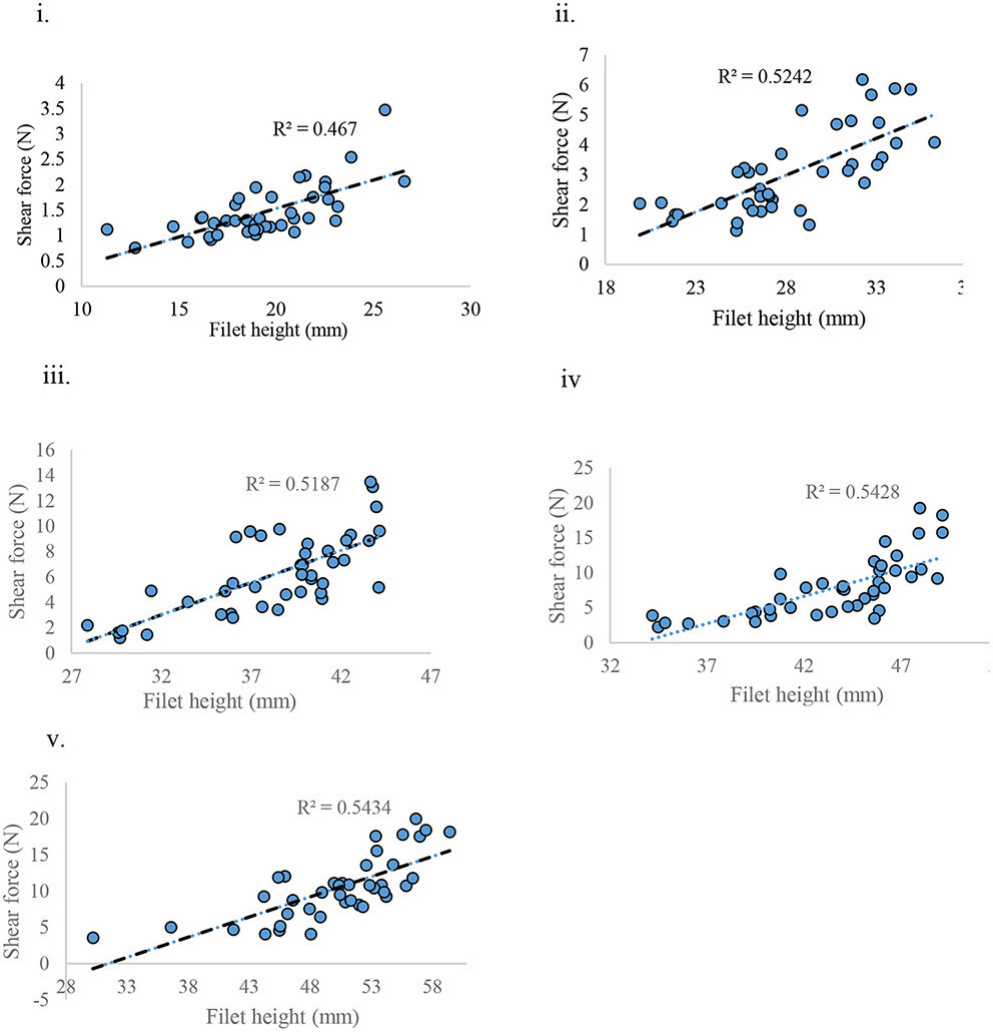
Shear force measured against filet height at various ages of bird grow-out cycle. **(i)** d 21, **(ii)** d 28, **(iii)** d 35, **(iv)** d 42, and **(v)** d 57.

When weight and height of the filet were correlated with shear force (**SF**), the weight of filet was found to have no correlation with SF (*R*^2^ = 0.001) (data not presented) whereas the height of the filet was positively correlated with SF (*R*^2^ = ~0.5) for each age group sampled (Figures 9i–v).

## Discussion

The use of stable isotope tracer to study muscle PT is a widely used methodology in humans, but only a handful of studies have been performed in poultry in studying mixed muscle PT. To our knowledge, this is the first study that evaluated collagen PT utilizing stable isotope technique in poultry species. Isotopes can be given either using primed constant infusion method, which requires a continuous tracer infusion for several hours, or through flooding method (bolus injection) which has recently been introduced without much difference in the end results regardless of the methods followed (14). The flooding technique measures the rate of protein synthesis in tissue by introducing large quantities of unlabeled amino acid along with tracer amino acid in blood to create an equilibrium in isotopic enrichment of the free amino acid in plasma and tissue compartments (15). In animals, the use of flooding method of tracer is more popular because of relative difficulty of constant infusion method. Flooding can be completed within few minutes to an hour depending upon the size of the animal that is being infused. The current study utilized the flooding method of tracer infusion in birds using brachial vein of bird.

Reports on few studies conducted in poultry that measured FSR in mixed muscle in *P. major* in broiler aged 2–4 weeks found progressive and significant decrease in FSR values (22.4–12.5%/d) (16). This decreasing trend in FSR with increasing age of the broiler was also true for collagen protein in *P. major*, as observed in the current study. The d 21 FSR value for collagen synthesis (~4.32%/d) found in this study was smaller as compared to mixed muscle FSR values reported in other studies (~12%/d) (16, 17). Collagen synthesis rates in skeletal tissue observed at d 21 was found to be similar (~5%/d) to that in another study conducted on 1 month old rats (18). Collagen synthesis in human skeletal muscle was found to be 0.016 ± 0.002% h^−1^ for young men (28 ± 6 y) and 0.023 ± 0.002% h^−1^ in elderly men (70 ± 6 y) (9). These values obtained in humans were similar to collagen protein synthesis rates in *P. major* muscle of broiler (>5 weeks) as reported in this current study.

Changes in collagen degradation can play a major role in regulating collagen mass in any tissue. The FSR and FDR values of I-collagen in *P. major* were higher in younger broiler and decreased over the age of bird. The difference in FSR and FDR values at each age sampled were greater as the broiler aged, indicating higher accretion of I-collagen occurring in maturing broiler. The difference between FSR and FDR values for S-collagen showed an increasing trend with age, similar to that of I-collagen until d 42. On d 57, higher FDR than FSR rate for S-collagen was observed. This elevated FDR of S-collagen observed at d 57 could be due to its true degradation occurring in *P. major*, or due to conversion of S-collagen into I-collagen (19). Of note, the percent accretion for I-collagen was less than that of S-collagen in birds from 21 to 42 days (3.98%/d for I-collagen vs. 8.72%/d for S-collagen). This still indicates that the daily accretion of I-collagen was markedly higher than S- collagen on pool size basis in *P. major*, as the quantity of I-collagen measured (≥3-folds) was higher than S-collagen at each age. Increased I-collagen pool size with increasing age of the broiler potentially indicated higher availability of substrate (collagen fibrils) for crosslinking resulting in the toughness of muscle (20). Results depicted that amounts of total collagen were ~4% at d 21 and was as high as ~11% at d 57 per mg of *P. major* (as is basis). Further, I-collagen was higher for myopathy affected broiler than non-myopathy ones. This higher collagen content in maturing broiler could be associated with myodegeneration of muscle fibers and subsequent replacement of muscle specific protein with collagenous tissue. The increment of collagen content in maturing was also supported by micrograph results where relative increase in non-fiber space per unit area of muscle tissue (~15–20% increment per mm^2^ after d 35 compared to non-fiber space existed at d 21) was observed.

Meat texture is largely affected by the amount and composition of intramuscular connective tissue (IMCT) (21). Considerable variation could exist in the expression of IMCT during the growth of animal, depending upon the functional characteristics of muscle (22). Studies done in bovine species show that perimysial collagen content is mostly responsible for the toughness of meat (23). This was also valid in broiler (particularly myopathy affected), with histomicrographs exhibiting relatively higher deposition of collagenous tissue and probable crosslinking of perimysial collagen fibrils occurring in *P. major* (Figures 7, 8). The MT staining utilized in this study has been a preferred method for better visualization for collagen in skeletal muscles (24). Micrographs revealed greater degeneration of muscle fibers occurring as early as d 21 in myopathy-affected broiler resulting in fibrosis of muscle tissue. Collagen protein is highly expressed in pathophysiology of fibrosis (25). Polyphasic myodegeneration was also evident in another study conducted in a fast-growing broiler line (4). In maturing broiler, *P. major* muscle developed toughness in muscle texture, and thus greater SF values and higher manifestation of WB myopathy was evident. Filet which had greater height indicated that those broilers were relatively more affected with WB myopathy condition as compared to other broiler of same age (26).

**Figure 10:**
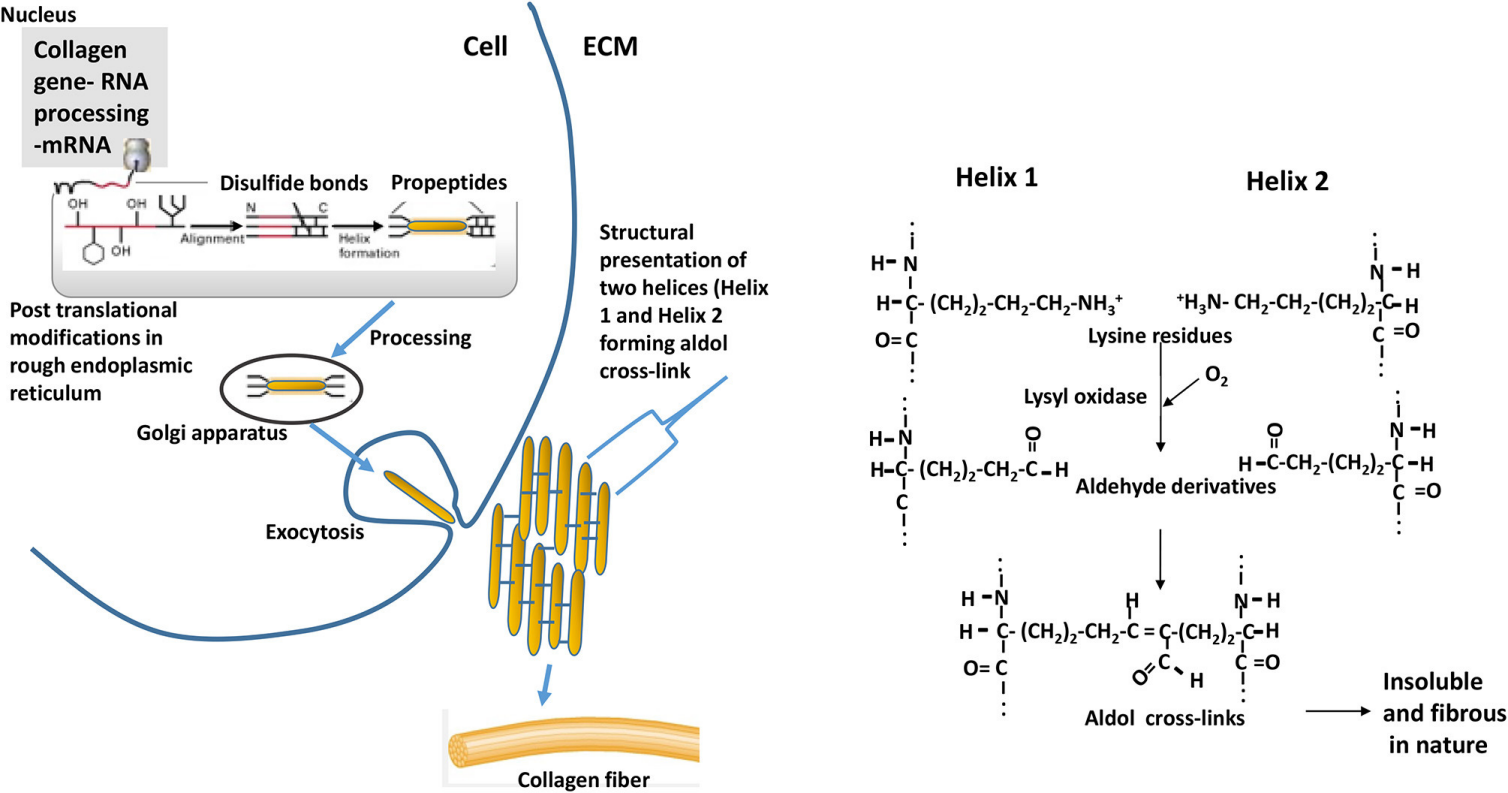
Events depicting the biosynthesis of fibrous collagens. Pre-procollagen polypeptides (α-helical region colored red) synthesized in nucleus go through events of post-translational modifications (hydroxylation and glycosylation) of certain proline and lysine residues to 4-hydroxyproline, 3-hydroxyproline, hydroxylysine, galactosylhydroxylysine, and glucosylgalactosylhydroxylysine. Catalyzing enzymes responsible for hydroxylation are prolyl 4-hydroxylase, prolyl 3-hydroxylase, and lysyl hydroxylase; whereas galactosyl transferase and glucosyltransferase are involved in glycosylation. Disulfide bonds formation (at C-terminal) among three procollagen polypeptides results in initiation of triple helix structure. Further processing of helix occurs in golgi apparatus, and then processed helix is exocytosed to extracellular matrix (ECM). Helices align laterally (presented as Helix 1 and Helix 2 in the schematics) and interact forming aldol cross-links between two lysine (or hydroxlysine) residues to form collagen fibrils. ECM = extracellular matrix. The reaction is catalyzed by enzyme lysyl oxidase. Adapted with modifications from Lodish et al. (27).

Intervention strategies for reducing the collagen formation in tissues need understanding biochemical processes involved in the synthesis of collagen. Schematics of the collagen synthesis is shown in Figure 10. Mechanistic targets to reduce the excess deposition of cross-linked collagen in ECM would be either reducing intracellular synthesis or increasing intracellular degradation of collagen peptides especially at earlier grow-out age of bird as more synthesis is occurring during that age. Collagen peptides are formed by repeating motif of glycine-proline-X (X, being any amino acid) in a triple helical structure, and the lysine residues are pre-dominant amino acid in forming aldol cross-links (Figure 10). Dietary modulations affecting the concentrations of these major amino acids can alter the collagen synthesis or rate of formation of cross-linkages. Intracellular degradation of collagen peptides can be targeted by decreased expression or production of prolyl or lysyl hydroxylases as these enzymes are involved in post-translational modifications of polypeptide chain and providing ultimate stability to pro-collagen. Another approach would be enhancing the extracellular degradation of collagen through extracellular matrix proteinases (**ECM**) proteinases after collagen fibrils are exocytosed. The ECM proteinases are mainly matrix metalloproteinases (MMPs), upon their activation initiate the proteolytic degradative pathways (28). Further, MMPs are also thought to have regulatory roles on muscle growth and repair. Collagenases of MMPs family are MMP-1, MMP-8, MMP-13, and MMP-18; these collagenases are involved in cleaving native helical structure of collagen types I, II, and III. As discussed earlier, the stabilization of collagen fibrils is provided by covalent cross-links, which are formed by lateral interaction of two helices by conversion of lysine residues to aldehyde derivatives (Figure 10). This formation of aldol cross-links is mediated by lysyl oxidase (27) which can be another target biomolecule to intervene *P. major* WB myopathy issue.

Overall, the findings in this study suggest that there is marked age related changes in collagen metabolism for both S- and I- collagen types in *P. major* muscle. Slower FSR values for I- collagen in *P. major* at the later age of bird could have evolved due to adaptive physiological feedback mechanism of bird against further synthesis and deposition of I-collagen in extracellular matrix, as the amount of collagen already exceeded the supportive role of fibrillar collagen. Since hardness of the muscle is governed by the overall extent of crosslinking of collagen fibrils in extracellular matrix, presence of higher amount of collagenous tissue in IMCT could potentiate substrate availability for collagen crosslinking. These preliminary while exploratory findings suggest that the mechanisms which enhance higher degradation of excessively accumulated extracellular collagen proteins, particularly I-collagen, in *P. major* in maturing broiler can be exploited as a viable intervention to reduce substrate availability for collagen crosslinking.

## Data Availability Statement

The datasets generated for this study are available on request to the corresponding author.

## Ethics Statement

Institutional Animal Care and Use Committee (IACUC) of University of Arkansas, Protocol # 17080, in compliance with Public Health Service Policy on Humane Care and Use of Laboratory Animals (PHS Policy), the USDA Animal Welfare Act and Regulations (AWAR), the institutional Animal Welfare Assurance, and the University Policy on Animal Care and Use approved husbandry practices and welfare guidelines performed in experiment.

## Author Contributions

PM wrote the manuscript. All authors contributed to the design of the study, performed the critical discussion of the results, and reviewed the manuscript.

### Conflict of Interest

The authors declare that the research was conducted in the absence of any commercial or financial relationships that could be construed as a potential conflict of interest.
